# Supplementary material for: Polymorphisms in Pfkelch13 domains before and after the introduction of artemisinin-based combination therapy in Southwest Nigeria
Source: PLoS One. 2025 Mar 31;20(3):e0316479. doi: 10.1371/journal.pone.0316479 (PMC11957316; doi:10.1371/journal.pone.0316479)

Samples: 13241
Bases: 1050
Average spacing: 13

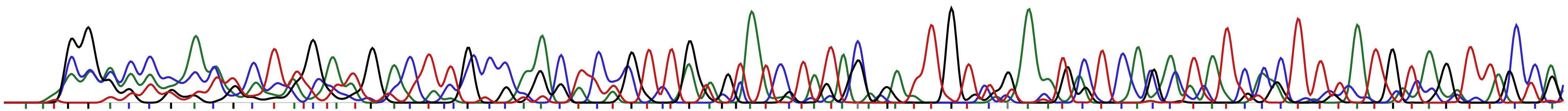

10 20 30 40 50 60 70 80

A A T G G A C C G A C G C T A T C T A T G A C T C C G C C A A C T C T G T C T G A G G A T C T A T A - A G A T T G T C - A A T C T C A C C T A T C C C T T T A T G T A G T T C C A

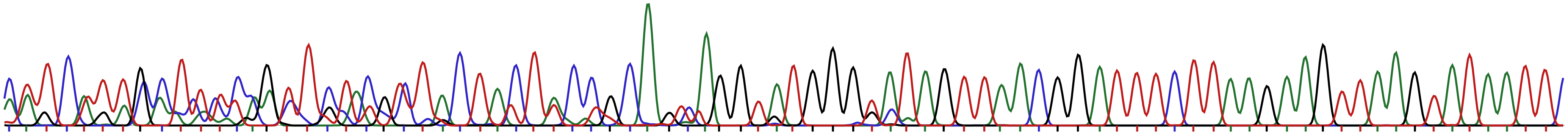

90 100 110 120 130 140 150 160 170

C A T C T T T G C T T T C A G T T C T C G C T A C T A C T T C C G C A G C A G G T A T G G G T A T A G T T A A C G G A T T T C T T A A G A A G T T A A G T A T A A T T

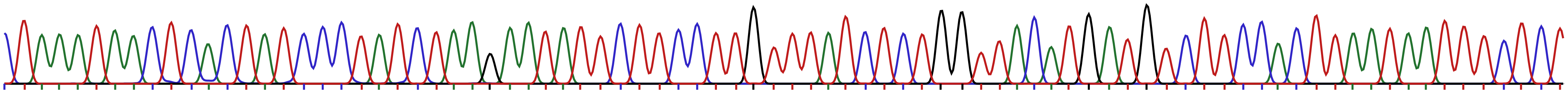

180 190 200 210 220 230 240 250

C T A A A T A A C T C A C T A T C C C T A T C T A A G A A T A T T C T T C C T T G T T T A T C T C T G G T T A C A T G A T G T C T T C C A C T T A A T A A T T T C T C T

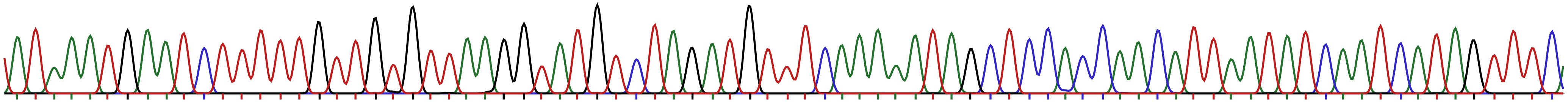

260 270 280 290 300 310 320 330

A T A A A T G A A T C T T T T T G T T G T G T T A A G G T A T G T C T A G A T G T T T C A A A A A T A G C T C C A C C A A C A T T A A T A T C A A T C A T A G T T T C

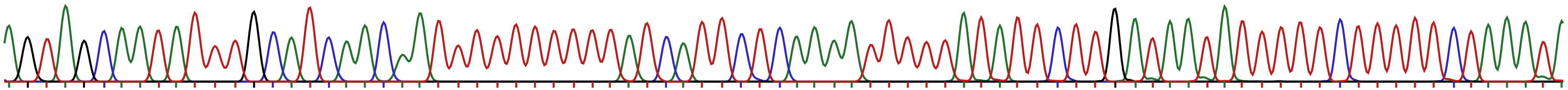

340 350 360 370 380 390 400 410 420

A G T A G C A A T A T T T G C A T C A A C A A T T T T T T T T T T A T C A T T C T C A A A A T T T T T A T A T T C T T G A T A A T A T T T T T C T T T T T C T A A A T A

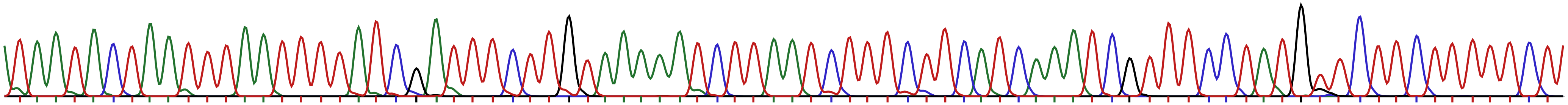

430 440 450 460 470 480 490 500

T A A T A C T A A T T T T A A T T T T A T C G A T T T C T T G T A A A A A T C T T A A T C T T T C T T C A T C A A A T C G T T T C C T A T G T T C T T C T T T T T C T

Samples: 13241
Bases: 1050
Average spacing: 13

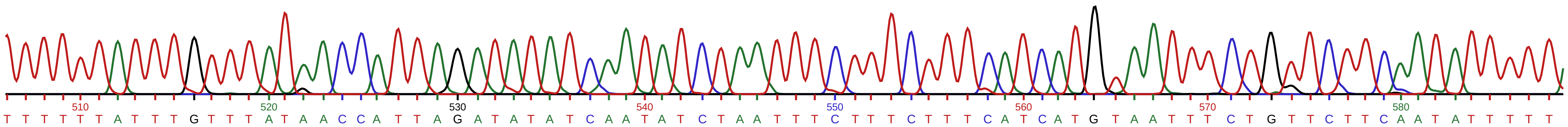


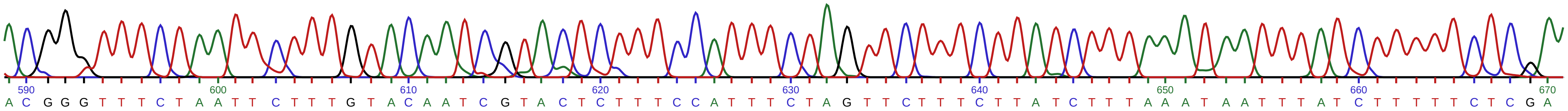


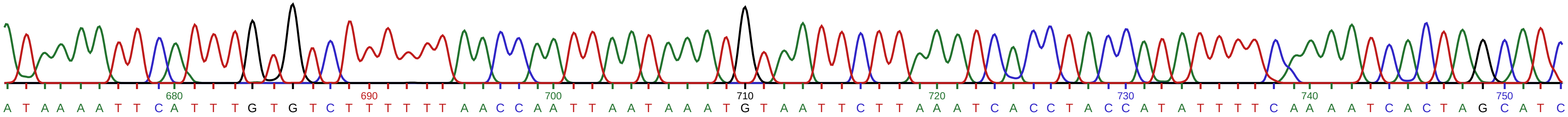


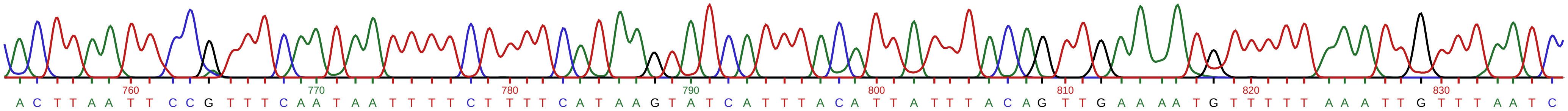


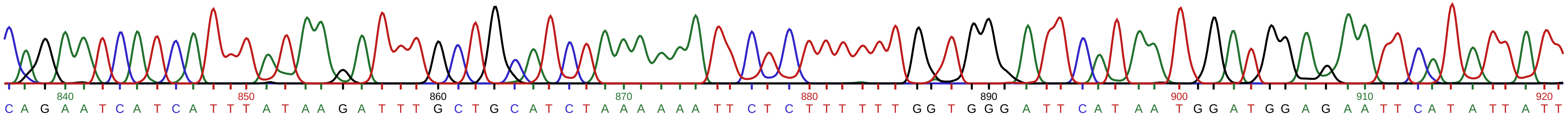


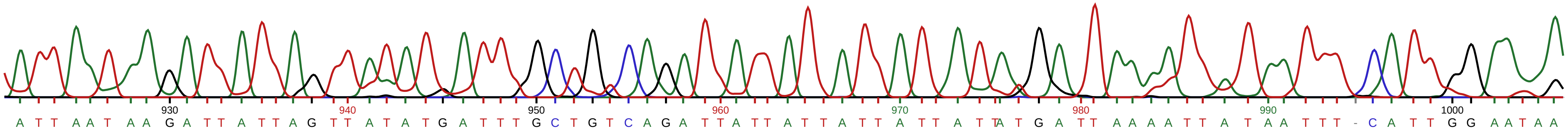

Samples: 13241
Bases: 1050
Average spacing: 13

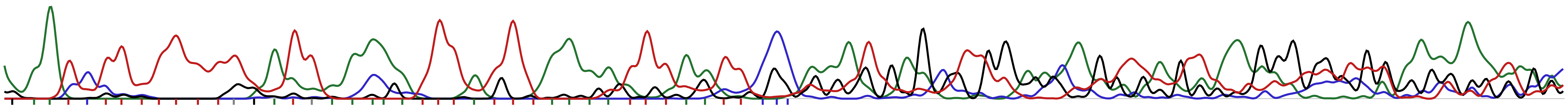

1010 1020 1030 1040

G A A T C T T T T T T T - G A T - A A A A A T T T A T T T A A A A T T T A A T T C C C

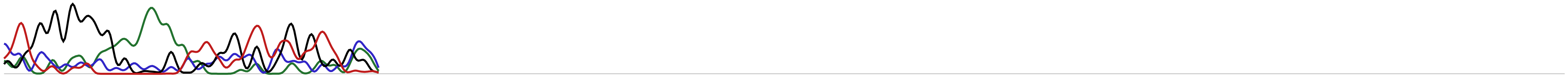

Supplement: Supporting information 2 — (ZIP) [file pone.0316479.s002.zip › 063KN2R_PREMIX_Plate_KELCH2_G03.pdf]
